# Identifying Defects in Aerospace Composite Sandwich Panels Using High-Definition Distributed Optical Fibre Sensors

**DOI:** 10.3390/s20236746

**Published:** 2020-11-25

**Authors:** James A. Mills, Andrew W. Hamilton, David I. Gillespie, Ivan Andonovic, Craig Michie, Kenneth Burnham, Christos Tachtatzis

**Affiliations:** 1 Lightweight Manufacturing Centre, Department of Design, Manufacturing and Engineering Management, University of Strathclyde, Glasgow G1 1RD, UK; 2 Department of Electronic and Electrical Engineering, University of Strathclyde, Glasgow G1 1RD, UK; andrew.w.hamilton@strath.ac.uk (A.W.H.); david.gillespie@strath.ac.uk (D.I.G.); i.andonovic@strath.ac.uk (I.A.); c.michie@strath.ac.uk (C.M.); christos.tachtatzis@strath.ac.uk (C.T.); 3 Collins Aerospace, 1 Dow Avenue, Prestwick International Aerospace Park, Ayrshire KA9 2SA, UK; 4 Advanced Forming Research Centre, Department of Design, Manufacturing and Engineering Management, University of Strathclyde, Glasgow G1 1RD, UK; kenneth.burnham@strath.ac.uk

**Keywords:** HD-FOS, OFDR, aerospace, thermal analysis

## Abstract

Automated methods for detecting defects within composite materials are highly desirable in the drive to increase throughput, optimise repair program effectiveness and reduce component replacement. Tap-testing has traditionally been used for detecting defects but does not provide quantitative measurements, requiring secondary techniques such as ultrasound to certify components. This paper reports on an evaluation of the use of a distributed temperature measurement system—high-definition fibre optic sensing (HD-FOS)—to identify and characterise crushed core and disbond defects in carbon fibre reinforced polymer (CFRP)-skin, aluminium-core, sandwich panels. The objective is to identify these defects in a sandwich panel by measuring the heat transfer through the panel thickness. A heater mat is used to rapidly increase the temperature of the panel with the HD-FOS sensor positioned on the top surface, measuring temperature. HD-FOS measurements are made using the Luna optical distributed sensor interrogator (ODISI) 9100 system comprising a sensor fabricated using standard single mode fibre (SMF)-20 of external diameter 250 μm, including the cladding. Results show that areas in which defects are present modulate thermal conductivity, resulting in a lower surface temperature. The resultant data are analysed to identify the length, width and type of defect. The non-invasive technique is amenable to application in challenging operational settings, offering high-resolution visualisation and defect classification.

## 1. Introduction

The aerospace maintenance, repair and overhaul (MRO) market was valued at $81.9 billion in 2019 and is projected to rise to $116 billion by 2029 [1]. Aerospace MRO companies face significant challenges in meeting the demand as traditional inspection methods rely on manual processes and discrete analysis equipment that are labour intensive and consequently slow [2]. There is growing pressure within the MRO sector to improve the number of composite components that are repaired rather than replaced, and to reduce the number of inspections of a given component to fully capture all defects, thus optimising current processes and reducing costs.

**Figure 1:**
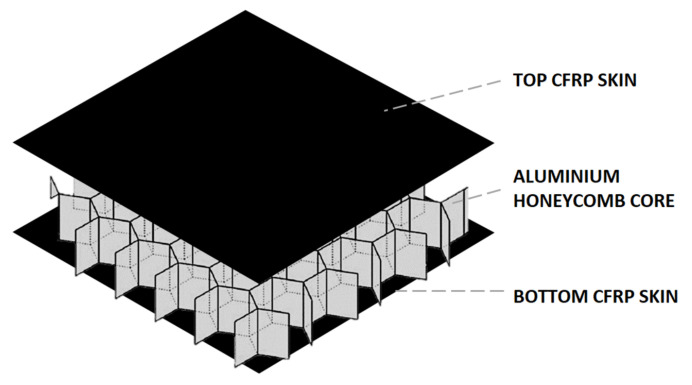
Structure of composite sandwich panel with two outer skins of carbon fibre reinforced polymer (CFRP) and inner core of aluminium honeycomb.

Currently, composite components represent 50% of the weight of commercial air frames with sandwich panels most often used for nacelles, rudders, elevators and wing-lets [3]. Aerospace sandwich panels typically consist of two outer layers (or skins) over a lightweight core, a combination proven to offer an excellent strength-to-weight ratio; although foam cores are used, aluminium honeycomb cores are preferred. Figure 1 depicts a carbon fibre reinforced polymer (CFRP) sandwich panel with an aluminium honeycomb core. The top and bottom skins are typically bonded to the core using an adhesive film [4].

The paper explores the use of high-definition fibre optic sensing (HD-FOS) for the detection of defects in aerospace-grade composite sandwich panels. Of particular interest are crushed core and disbond defects that occur in CFRP-skin, aluminium-core sandwich panels either during manufacture or in-service. The technique offers a more agile solution for MRO settings. The use of HD-FOS for the detection of defects within composite sandwich panels is novel and has huge potential for academic and industrial applications.

It is important to stress the distinction between non-destructive inspection (NDI) and non-destructive testing (NDT) in order to position the reported development. NDI methods can identify if a defect is present; NDT methods permit certification of a component or repair.

A traditional NDI method for composite components is acoustic testing (also known as tap-testing), during which a testing hammer lightly taps the component surface to induce an acoustic response from the structure [5]. Healthy regions will produce an even tone, whilst defective areas will create a duller tone owing to the damping of the resonance [6]. This method has been proven effective for identifying the following types of defect:

- Disbond—a break in the resin between the composite skin and the aluminium core allowing an air gap to form.
- Crushed core—compression of the aluminium core.

An NDT procedure must be undertaken to fully characterise the defect after tap-testing, typically taking the form of an ultrasonic-enabled inspection [7]. High-frequency sound waves are passed through the object and the resultant ultrasonic response provides insight on the integrity of the material [8]. In well-bonded structures, the transmitted acoustic energy will be attenuated by the combination of components and adhesive. Transmission through a defective section of the sandwich structure under inspection modulates the amplitude of the signal, manifesting in the received acoustic energy.

## 2. Related Work

Ultrasonic inspection can be performed with pulse-echo [9], pitch-catch [10] or through transmission ultrasound (TTU) [11] techniques; the former uses one probe to generate and receive the sound signal whilst the latter two require a separate receiver. Ultrasonic pulse-echo inspection can be highly sensitive to surface defects but is less effective for identifying and characterising crushed core defects [7], a result of the largely porous nature of the aluminium honeycomb structure. Pitch-catch can be used to detect defects in sandwich structures, however the method is applicable for large defects and not suitable for uneven surfaces [12]. TTU is capable of providing defect information, however, in order to inspect complex geometries, dexterous robotic solutions are required [13], which are often difficult to deploy in an MRO setting.

Inductively coupled ultrasonic sensors, an alternative use of ultrasonic technology, facilitate in-situ measurement of defects in composite components. The method requires two components: a thin sensor located either on the surface or embedded in the component and a hand-held data collector. The sensor requires no battery resulting in a profile thickness of 1 mm. The method also removes the possibility of human error that may be a factor in traditional ultrasound techniques. However, the disadvantages are the quantity of in-situ sensors required to establish a true mapping of all component defects and the sensors’ limited capability to detect defects in thick materials or complex geometries [14].

A variety of other inspection methods have been explored in industrial environments including radiography, laser shearography, thermography and eddy current detection [15,16,17,18]. In radiography, defects are detected by penetrating electromagnetic waves through the component, which are captured by a detection unit on the reverse side of the material. The presence of a defect results in a reduction in the intensity of the detected radiation in comparison to a non-damaged region, visualised on the generated radiograph. Radiography is particularly useful for the detection of cracks and water ingress with a limited capability to identify delaminations. Conversely, it is ineffective in identifying disbonds and barely visible impact damage (BVID) [7], limited by some of the same issues as are encountered with acoustic methods in MRO settings, viz., restriction in the practical location of the receiver unit.

Laser shearography executes a comparison of the same component in two states, under-load and free-standing. The applied laser light produces surface excitations which are compared by means of an interferometer to reveal any defects in the material. Shearography has been successfully used to detect surface defects in honeycomb sandwich panels, such as delaminations and disbonds, but has been shown to be less effective at detecting defects in the core. The resolution of the defects detected is lower than that achievable with ultrasound [12]. In eddy current detection, the conductivity of carbon fibre and the resistance of resin systems are used to determine variations in the impedance across the component, which help to identify the locations of defects. A prior knowledge of the resistance of the undamaged component is the reference against which measured increases across the CFRP indicate the presence of a defect [18]. The location and size of the defect may be determined by monitoring the resistance across multiple routes (fibres) within the material. Eddy current mapping is limited to the detection of surface defects such as delaminations and surface cracks but cannot identify crushed core damage.

Thermography is implemented through measurements of the surface temperature of a component to determine the location of a defect by identifying the changes in the local thermal conductivity, carried out using transient or lock-in techniques. Transient thermography measures the heat signature of the component as it cools using an infrared camera. Lock-in thermography uses a series of heat pulses at a specific frequency. The input and output heat excitations are subsequently compared, with out-of-phase readings indicating the presence of defects [17]. Thermography has been proven to be a viable approach for the detection of delaminations and disbonds. However, a series of readings is required under consistent measurement conditions in order to capture accurate thermographic responses, which is a challenge in MRO settings where the component may be under vacuum conditions. However, contact temperature sensors and heater mats have been demonstrated as an NDI method suitable for the MRO environment [19].

### 2.1. Rayleigh Backscattering Sensors

Optical fibre distributed temperature and strain sensors (ODTSS) systems have been studied extensively for a wide range of structural integrity monitoring applications [20,21,22]. Brillouin-scatter-based systems have been demonstrated to provide measurement sensitivities in the order of 2.2 ${}^{\circ }$C and ±44μϵ over distances that extend to several tens of kilometres [23]. Rayleigh-scatter-based optical time domain reflectometry (OTDR) measures the losses within an optical fibre as a function of distance [24]. OTDR has often been applied over extended lengths of fibre (km) at a spatial resolution in the order of metres depending upon the optical pulse width [25]. Losses can be induced by temperature, strain or both; those attributed to temperature variations have a low sensitivity in the region of 5 ${}^{\circ }$C [26].

Fibre Bragg gratings (FBGs) also measure changes in backscatter to identify changes in physical phenomena. FBGs are written into an optical fibre and act as a filter, designed to only reflect certain wavelengths. This results in a change in the reflected wavelength, owing to changes in temperature or strain within the optical fibre [27]. Multiple FBGs can be embedded into the same optical fibre and can be interrogated individually by either time or wavelength division multiplexing. However there is a limit to the number of gratings that can be multiplexed, typically ≈100 [28], although some research has postulated that a value of up to 1000 gratings is theoretically possible [29]. Optical frequency domain reflectometry (OFDR), implemented by frequency chirped sources, provides higher spatial resolution (in the region of mm) and temperature sensitivity in the order of (0.1 to 1 ${}^{\circ }$C) [30].

The main limitation in the sensitivity and accuracy of Rayleigh-scatter-based measurements arises from the fact that the scattered light captured by the fibre and returned to the detector is low. Higher scattering fibres such as liquid filled hollow core fibres have been shown to increase the signal-to-noise ratio (SNR), but require specialist manufacturing techniques and are therefore costly. The sensitivity of standard single-mode fibre can be significantly enhanced by UV exposure, yielding improvements in sensitivity of the order of 1 mC [31].

Changes in both temperature and strain affect the length of the fibre and the core refractive index and lead to cross sensitivity between measurands [32]. In order to decouple the two parameters, a combination of Brillouin scatter and Rayleigh backscatter can be measured using OFDR. The scheme was reported to yield a measurable range of 92 m and a spatial resolution of 50 cm with accuracies of ±1.2 ${}^{\circ }$C in temperature and ±15μϵ in strain [33].

The potential for the utilisation of sensors based on standard SMF28 single mode fibre, interrogated using a coherent OFDR (Luna Inc) to detect temperature changes along the length of an optical fibre, is explored here. Temperature variations of the order of 1 ${}^{\circ }$C, compatible with the performance of the OFDR, are sufficient to detect defects within composite structures.

### 2.2. Industrial Applications

HD-FOS has been used in a variety of industrial applications. The strain detection capabilities are aligned with the needs in structural health monitoring such as fatigue analysis of wind turbine blades [34]. Optical fibres have also been embedded in pressure vessel over-wrapping, allowing damage detection during both the manufacturing process and in-service operation [35]. The temperature detection capabilities of the technology have also been utilised in the nuclear industry for reactor monitoring and in the steel industry for surface temperature mapping [36]. Further, the avionics industry has integrated HD-FOS for fault-finding and trouble-shooting of on-board telecommunications [37]. Extensive work has been carried out using this method on composite laminates for the monitoring of strains during manufacture and service life [38]. However, its use for detecting defects in composite sandwich panels has not been demonstrated to date and has the potential to characterise crushed cores and disbonds in combination with thermal transmission analysis.

## 3. Methods and Materials

**Figure 2:**
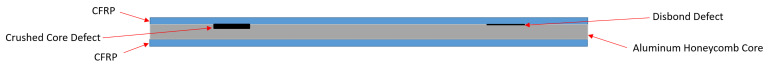
Cross-sectional view of the sandwich panel with highlighted defect types.

**Figure 3:**
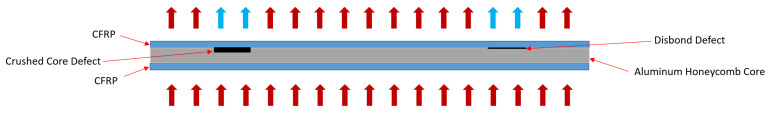
Cross-sectional view of the sandwich panel with heat transfer from the heater mat.

**Figure 4:**
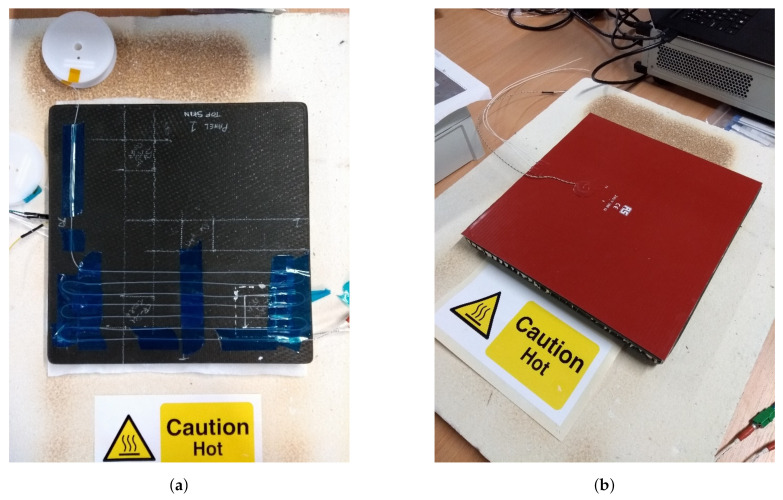
Experimental set-up. (**a**) Top-skin of the sandwich panel with the high-definition fibre optic sensing (HD-FOS) sensor located on the CFRP surface. (**b**) Heater mat located on the bottom-skin of the sandwich panel, with integrated type J thermocouple.

**Figure 5:**
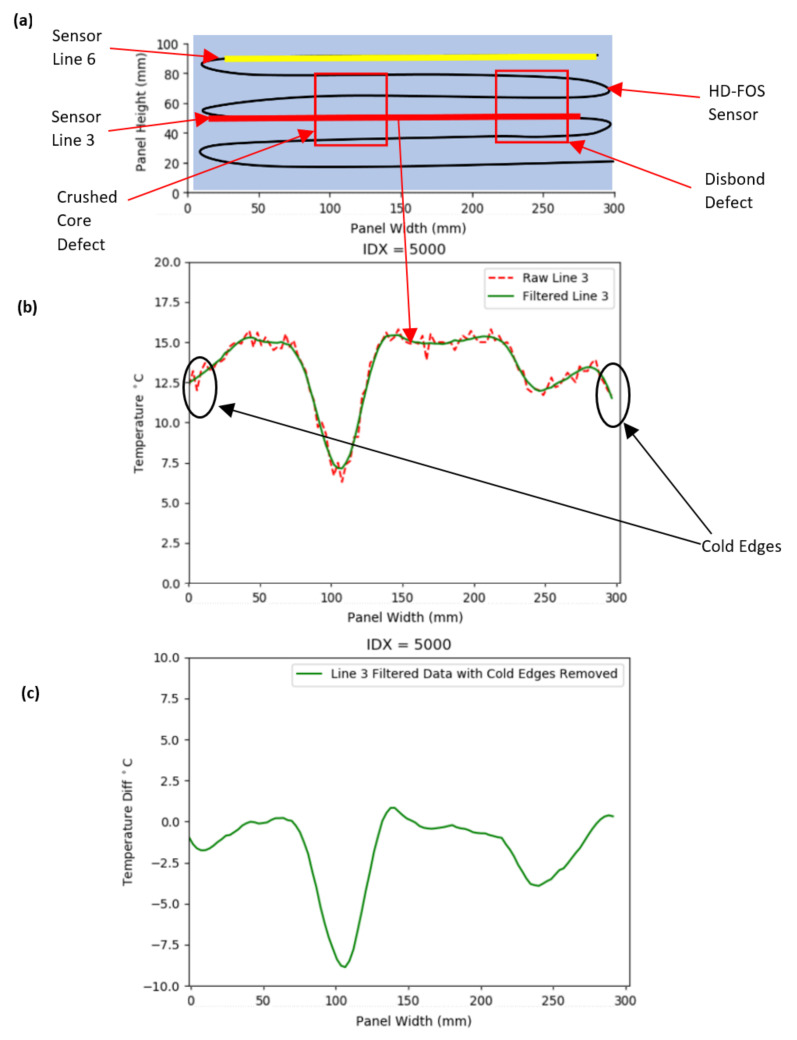
(**a**) Annotated segment of the composite sandwich panel top surface with defects and HD-FOS sensor shown. (**b**) Sensor Line 3 raw data and Savitzky–Golay filtered data comparison with cold edges highlighted. (**c**) Sensor Line 3 Savitzky–Golay filtered data with cold edges removed.

To evaluate the suitability of HD-FOS for non-destructive inspection of honeycomb lamina in aerospace MRO, an experimental schedule was produced to apply this technology to representative components. A 300 × 300 mm sandwich panel was constructed with two 4-ply (0/90/0/90), five-harness, satin weave CFRP laminates with an aluminium honeycomb 9.525 mm cell field core. The CFRP skins were vacuum bagged and cured. Aerospace CFRP is typically cured at 180° and 700 kPa for 2 h. Subsequently, an adhesive film was used to bond the CFRP laminate with the honeycomb structure. The CFRP skins had a thickness of 0.734 mm with an aluminium core thickness of 30 mm giving a total panel thickness of 31.468 mm. The sandwich panel configuration was selected as it is representative of the material construction used in aerospace-grade composite panelling. Two different defects were induced in the sample (Figure 2); the first a crushed core, with dimensions 38.1 × 38.1 mm, produced by impacting the aluminium honeycomb cells with a hand-tool prior to bonding with the CFRP skin. This represented a crushed-core defect that can occur during the manufacture or repair of a sandwich panel whilst undergoing autoclave curing. The pressure difference between the vacuum in the honeycomb structure and autoclave atmosphere initiates the lateral crushing action [39]. The defect can also occur in service as a result of hail storms or by tool-dropping during maintenance [40]. The second defect was a disbond, a feature at the bond between the aluminium core and the CFRP laminate. In order to emulate the defect, two stacked polytetrafluoroethylene (PTFE) inserts, with dimensions 38.1 × 38.1 mm, were placed between the CFRP laminate and the aluminium hex core to create a disbond. Figure 3 represents the theoretical heat transfer from the heater mat through the panel. The HD-FOS sensor was positioned along six parallel lines of the bottom 100 mm of the sandwich panel, at a 13 mm spacing between each, see Figure 4a. This was to maintain the minimum bend radius of the fibre whilst providing good resolution. High-temperature polyester (PET) tape held the sensor in place and covered the area of both defects and pristine areas, see Figure 5a.

The panel was heated evenly and rapidly on one side by an RS PRO 396 W, 240 V ac Silicone Heater Mat to 80 ${}^{\circ }$C. The heater mat had an inbuilt type J thermocouple, which provided temperature measurements and control capabilities to ensure an even and consistent heat delivery, see Figure 4b. The temperature uniformity of the heater mat was tested using a thermal imaging camera to ensure a homogeneous application of heat. The OFDR-based fibre-optic distributed temperature sensor was configured to provide measurements at a spatial resolution of 2.6 mm, giving 648 data points across the six lines at a frequency of 25/s. 73,018 sets of data providing spatially distributed temperature measurements over the test panel were acquired over a near 49 min period. The HD-FOS unit was controlled through the optical distributed sensor interrogator (ODISI) graphical user interface, which provided live readings and collected the data from the optical fibre. The sets of data were stored on a.csv file for analysis.

Given that the HD-FOS sensor measures changes in both temperature and strain, the experimental methodology for collecting the relevant data ensured only temperature changes were recorded. The fibre was held in position with aerospace-grade flash tape to set its position on the flat panel (see Figure 4a), and any insulation effect created by the tape was considered negligible. This process prevented the fibre from moving and ensured that no external strain could influence the collected data. Once fixed, and at ambient room temperature, the sensor was zeroed by subtracting the baseline values from the signal. The manufacturer-defined calibration procedure was followed to ensure the best results were attained. Given that the panel expands uniformly when heated, it can be assumed that the collected data is representative of the temperature change on the surface of the panel.

## 4. Results and Discussion

Figure 5b shows the raw measurement data from Sensor Line 3, shown in Figure 5a. The data was filtered using a Savitzky–Golay Filter, with a window length of 15 and a degree-3 polynomial, to smooth noise, selected as it provides excellent performance without loss of key data features, e.g., peaks and troughs, when compared with moving average filtering [41]. An example of the output of the filtering is shown in Figure 5b. The IDX (index) value represents the data set number, out of the total 73,018 data sets collected. A value of 5000 equates to 3 min and 20 s after the heater mat was turned on.

As a result of convection, the “cold” edges of the panel were more prominent than expected, potentially masking defects on the colour-mapping of the component. Thus, the uppermost sensor output (unaffected by defects), shown as “Sensor Line 6”, was used as a datum from which the temperature differences of the other five sensors were recorded. As shown in Figure 5c, the methodology successfully removed the cold edges from the data.

**Figure 6:**
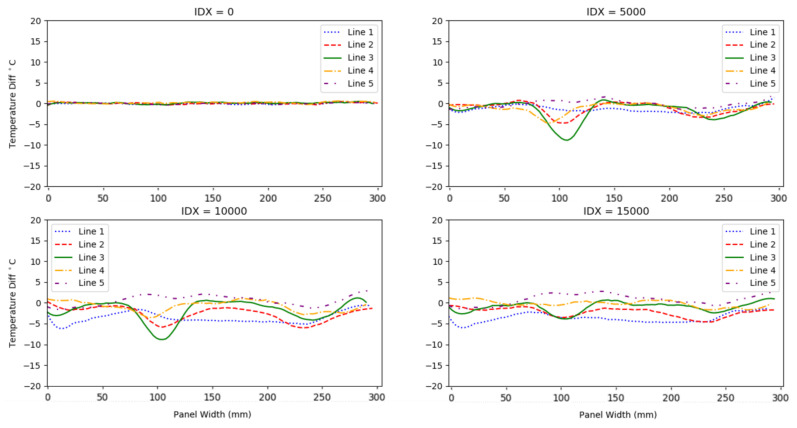
Graph series showing the temperature differences of Sensor Lines 1–5 from Sensor Line 6 (datum) at four points in the experiment. IDX (index) values indicate which data set is displayed.

In Figure 6, the temperature differences of the remaining five sensor outputs are displayed over a 10 min period. The sensor length is displayed on the x-axis, and temperature difference from a datum value of 0 ${}^{\circ }$C on the y-axis. A region of significantly lower surface temperature—a reduction of approximately 13 ${}^{\circ }$C from the datum—is visible in alignment with the position of the crushed core. The position is identified at 364 mm along the fibre, which corresponds to 86 mm along Sensor Line 2 (from the left). The second trough in temperature, which begins at 474 mm along the optical fibre corresponding to 195 mm along Sensor Line 2 (from the left), is less pronounced, nevertheless identifying the position of the disbond defect; the disbond induces a temperature difference of 6 ${}^{\circ }$C.

**Figure 7:**
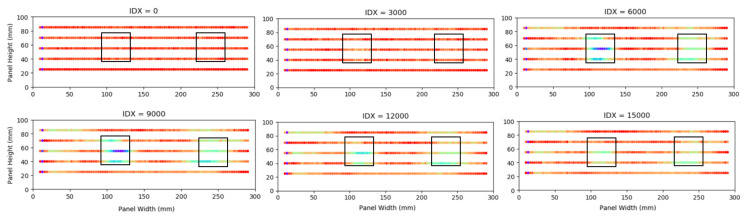
Colourmap graph series showing the temperature differences of Sensor Lines 1–5 from Sensor Line 6 (datum) indicating defect locations. Defect locations have been superimposed onto the graphs.

Figure 7 depicts the data on a colour map, highlighting the locations of the defects. The lengths and approximate widths of the crushed core and disbond defects were estimated from the data. The crushed core has a length of 39 mm and width of approximately 26 mm; the disbond has a length of 54 mm and an approximate width of 39 mm. The PTFE inserts used for the disbond were 38.1 × 38.1 mm and the crushed core area was approximately 38.1 × 38.1 mm. The derived area of the crushed core is accurate; however the disbond defect area is larger than expected, attributed to slippage between the PTFE inserts resulting in a larger thermographic silhouette.

The experimental analysis demonstrates that the technique has strong potential for detecting and quantifying internal defects within composite materials. Similar measurements can be made using conventional thermographic methods, however, in many instances access to the composite surface is challenging. The technique offers a viable route to detect defects with similar resolution as obtained from ultrasonic and other thermographic measurements. However, it is also amenable to implement in environments where integrating ultrasonic sensors or thermal cameras is challenging. The simplicity of the system allows for rapid set-up and analysis of data. In addition to providing a tool for inspection, distributed temperature measurements can offer deeper insights into the repair-curing process, enabling the potential for system control, e.g., to ensure a homogeneous cure.

Traditional defect detection methods are not effective in capturing the profile of honeycomb cores. The walls of the honeycomb are only fractions of a millimetre thick, creating issues for commonly used pulse-echo systems; the large pockets of air in the honeycomb result in the reflection of ultrasonic waves masking defect detection. HD-FOS offers a viable solution to overcome these limitations.

The implementation of the HD-FOS system in manufacturing or repair settings would increase the throughput of the process by reducing wastes associated with current industry procedures. The primary saving is in the number of defective components manufactured or repaired, with concomitant benefits with respect to other lean wastes including over-processing, transportation and wait periods. The optimisation of the inspection process results in time and financial savings, central to the return-on-investment decision in acquiring any inspection tool.

The novel use of the HD-FOS technique within an aerospace MRO setting offers the potential for rapid and accurate defect detection. The simple component set-up, easy-to-use graphical user interface and rapid data generation, coupled with excellent resolution and a responsive sensor profile, produces a solution with huge potential for further industrial and academic exploration.

## 5. Future Work

In order to increase the accuracy of the sensor readings and to negate the thermal impact of the PET tape, the optical fibre will be bonded to the surface of the CFRP skin. The solution will also be tested for rigour on more complex geometries for a range of defects including delaminations, water saturation and bottom-skin (heating side) defects.

## 6. Conclusions

In this paper we have reported preliminary evidence of the potential for HD-FOS-enabled temperature measurements to detect crushed core and disbond defects in composite sandwich panels. A 300 × 300 mm aerospace-grade test panel constructed with CFRP skins and an aluminium honeycomb core with two defects, a crushed core and a disbond, induced within the panel during manufacture was monitored. A thermal stimulus, from a bottom-surface mounted silicone heat mat with embedded type J thermocouple, was applied to reveal areas with poor thermal conductivity owing to defects. The thermal profile was detected with a top-surface mounted HD-FOS sensor secured to the panel. The sensor data, collected through OFDR, was subsequently processed to provide a colour map of a section of the test panel. A crushed core defect of 39 × 26 mm and a disbond defect of 54 × 39 mm were identified successfully. Initial results suggest that differentiation between the defects is possible given the recorded temperature differential on the panel surface attributed to each defect.

The paper has reported on the first use of HD-FOS for defect detection in composite sandwich panels and the findings offer a significant opportunity for further research. The technique is also applicable in a range of post-manufacture and post-repair inspection processes within industries such as oil and gas and automotive, offering advantages over currently used NDT methods based on ultrasound and infrared thermography. Future work will include bonding of the optical fibre to the CFRP to increase accuracy and more comprehensive evaluation in complex geometries. Characterisation of performance over a wider range of defects is required to quantify the capability to reliably differentiate between disbond and crushed core damage. There is scope to test the technique on defects such as water saturation, delaminations and bottom-surface flaws.

## Abbreviations

The following abbreviations are used in this manuscript:

BVID	Barely visible impact damage
CFRP	Carbon fibre reinforced polymers
FBG	Fibre Bragg grating
HD-FOS	High-definition fibre optic sensing
MRO	Maintenance, repair and overhaul
NDI	Non-destructive inspection
NDT	Non-destructive testing
ODISI	Optical distributed sensor interrogator
ODTSS	Optical fibre distributed temperature and strain sensors
OFDR	Optical frequency domain reflectometry
OTDR	Optical time domain reflectometry
PTFE	Polytetrafluoroethylene
SMF	Single mode fibre
SNR	Signal-to-noise ratio
TTU	Through transmission ultrasound
